# Coupling and Coordination in Gene Expression Processes with Pre-mRNA Splicing

**DOI:** 10.3390/ijms16035682

**Published:** 2015-03-11

**Authors:** Kewu Pan, Jimmy Tsz Hang Lee, Zhe Huang, Chi-Ming Wong

**Affiliations:** State Key Laboratory of Pharmaceutical Biotechnology, Department of Medicine, Shenzhen Institute of Research and Innovation, The University of Hong Kong, L8-43, 21 Sassoon Road, Pokfulam, Hong Kong, China; E-Mails: pankewu@hku.hk (K.P.); jimmylee@connect.hku.hk (J.T.H.L.); huangzhe@connect.hku.hk (Z.H.)

**Keywords:** pre-mRNA splicing, RNA surveillance, exosome, microRNA processing, mirtron, circular RNA

## Abstract

RNA processing is a tightly regulated and highly complex pathway which includes transcription, splicing, editing, transportation, translation and degradation. It has been well-documented that splicing of RNA polymerase II medicated nascent transcripts occurs co-transcriptionally and is functionally coupled to other RNA processing. Recently, increasing experimental evidence indicated that pre-mRNA splicing influences RNA degradation and *vice versa*. In this review, we summarized the recent findings demonstrating the coupling of these two processes. In addition, we highlighted the importance of splicing in the production of intronic miRNA and circular RNAs, and hence the discovery of the novel mechanisms in the regulation of gene expression.

## 1. Introduction

Most eukaryotic protein-coding genes contain introns. Human primary pre-mRNAs on average contain approximately 27 K nucleotides and 9 exons, but an average mature mRNA contains only 3.5 K nucleotides [1]. In other words, more than 85% of the nucleotides are intronic sequences which should be removed before the mRNA is being translated. The reason why cells waste so many resources to generate the “junk” during transcription remains a mystery. However, undoubtably, an effective system to recognize and remove introns is essential for preventing the production of abnormal proteins, which may function in a dominant negative manner and competitively inhibit the activity of their full-length native form [2].

**Figure 1:**
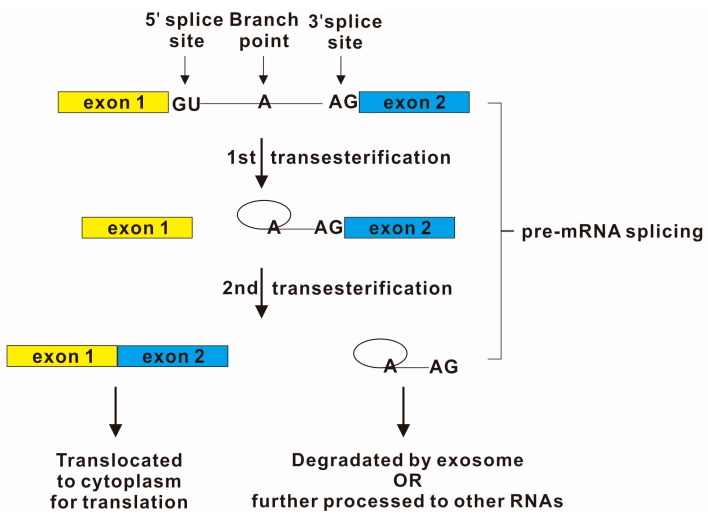
Pre-mRNA splicing includes intron exclusion and exon ligation. In most cases, introns start from the sequence GU as 5' splice sites and end with the sequence AG as 3' splice site. A highly conserved nucleotide A at the branch site located approximately 20–50 bases upstream of the 3' splice site. Lariat was considered as an unstable intermediate. Recent findings suggested that those intron products have unexpected long half-lives and are precursors for other RNAs such as miRNAs from mirtrons [6]. The factors determining the stability and fate of intron products are largely unknown.

Pre-mRNA splicing is a succession of two transesterification reactions (Figure 1). The reactions are catalyzed by the complex named spliceosome. Spliceosome is a complex comprised of both RNA molecules (e.g., small nuclear ribonucleoproteins) and proteins. Spliceosome is found throughout the entire nucleus [3], where transcription and many other RNA processing pathways take place. Spliceosome recognizes a donor splice site and an acceptor splice site that are located at the 5' and 3' end of intron, respectively. For the 5' splice site, the only highly conserved *cis*-elements are the proximal dinucleotide (GU) of the intron. However, for the 3' splice site, three separated *cis*-elements are required: the branch site, the polypyrimidine tract and the 3' splice site dinucleotide (AG). In brief, for the first trans-esterification reaction, the 2' hydroxyl group of the conserved adenosine at the branch site attacks the conserved guanine of the 5' splice site at the exon-intron junction. A 2'–5' phosphodiester bond is formed and the exon-intron junction is cleaved. A 2'–5' phosphodiester RNA lariat structure and a free 3'-OH (leaving group) at the upstream exon are produced. After the rearrangement of the spliceosome components, the second trans-esterification reaction begins with another nucleophilic attack. The 3'-OH end of the released exon attacks the scissile phosphodiester bond of the conserved guanine of the 3' splice site at the intron-exon junction. Finally, the two exons are ligated together and the intron is released as a stable lariat structure product [4]. The lariats need to be debranched by debranching enzymes before degraded or processed into useful RNAs such as intronic snoRNAs and mirtrons [4]. Intronic lariats will accumulate in the cytoplasm in the absence of Dbr1 enzymatic activity [5].

In addition to the 5' and 3' splice sites mentioned above, additional *cis*-elements named exonic/intronic splice enhancers or silencers can also influence the overall fidelity of pre-mRNA splicing [7,8,9]. An analysis focusing on mutations near splice junctions revealed that approximately 15% of disease causing mutations lead to RNA splicing defects [10,11]. With the advent of advanced strategies for predicting the effects of sequence variations on splicing and cryptic splice sites, more diseases caused by splicing defects will be explored [12,13,14]. Defects in pre-mRNA splicing are considered as the primary cause of many diseases, such as neurodegenerative diseases and cancers [15,16,17,18,19,20]. Hence, targeting pre-mRNA splicing could be a potential treatment for those diseases [5,21,22,23,24,25].

**Figure 2:**
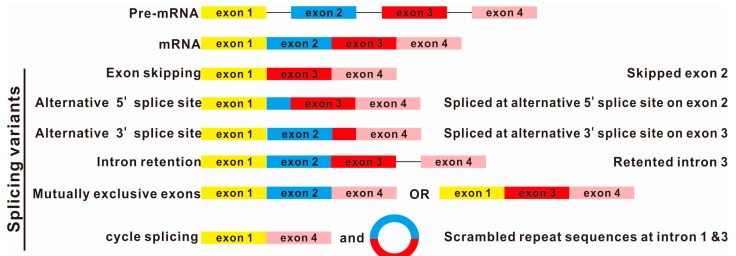
Many splicing variants could be formed from the same pre-mRNA by alternative splicing. Circular RNA, generated by splicing, is a new member of the splicing variants. Several mechanisms for the formation of circular RNAs have been proposed, including the circularization of exons, facilitated by the presence of adjacent repetitive sequence [33,34,35,36].

On the other hand, pre-mRNA splicing requires some degree of flexibility [26]. Exons and introns are either retained or removed to generate a diversity of splicing variants known as alternative splicing [27,28]. Alternative splicing is essential for regulation of gene expression and for increasing the proteome complexity. For example, a premature stop codon is introduced by alternative splicing that suppresses the expression of the gene by degradation through nonsense-mediated decay (NMD) during cytoplasmic translation [29]. In addition, alternatively spliced mRNA variants can produce protein isoforms with altered amino acid sequences and domains resulting in changes in enzymatic activity, cellular localization and/or binding partners [1]. Therefore, alternative splicing is considered to be the most important source of structural and functional diversity at the protein level. It is estimated that about 95% of transcripts from multi-exon genes undergo alternative splicing, some instances of which occur in a tissue-specific manner and/or under specific cellular conditions [30,31]. There are four main types of alternative splicing events (Figure 2), including exon skipping, intron retention, alternative 3' splice site and 5' splice site selection [27]. More complex alternative splicing events such as mutually exclusive exons, exon/intron scrambling, alternative promoter usage and alternative polyadenylation are less frequent [27,32].

Although splicing is tightly regulated [37,38,39,40], several lines of evidence suggested that the splicing of many pre-mRNAs is suboptimal [41] and that unspliced nascent transcripts and aberrant splicing intermediates are detected, especially when the intracellular RNA degradation activities are inhibited [42,43,44,45,46,47,48]. The recognition and degradation of the unspliced/mis-spliced transcripts and the excised introns become very crucial steps to maintain proper cellular growth and even survival. In this review, we summarized recent findings in coupling and coordination in gene expression processes with pre-mRNA splicing. The “by-products” generated from splicing escaped from RNA degradation were also discussed.

## 2. Splicing and Nuclear RNA Surveillance

So far, most studies are focusing on the recognition and degradation of unspliced mRNA by nonsense-mediated mRNA decay (NMD) [49,50,51]. NMD is an important RNA surveillance system that functions to detect and degrade RNAs with premature stop codon and prevent the expression of erroneous or truncated proteins in cytoplasm. A typical branchpoint usually harbors a translation termination codon without proper splicing. It remains at the unspliced RNAs and triggers the activity of NMD [46]. Therefore, the stop codon within splicing signal provides an important role to guarantee the cytoplasmic degradation of unspliced transcripts by NMD.

Nevertheless, a number of observations bring to the idea that nuclear RNA surveillance system not only plays a key role in eliminating the aberrant unspliced transcripts and splicing intermediates, but also directly involves in the regulation of the splicing process. Firstly, most of the unspliced mRNAs are trapped in the nucleus [52,53]. Secondly, unspliced transcripts and splicing intermediates are hardly detected in wild-type cells unless nuclear RNA surveillance is inactivated [42,43,44]. Thirdly, certain nuclear exosome components are recruited to intronic regions of transcribing genes [54,55,56]. Fourthly, a number of RNA binding factors, such as shuttling Ser-Arg-rich (SR) RNA-binding proteins and cap binding complex (CBC), which are recruited cotranscriptionally and exhibit physical or genetic interactions with nuclear RNA surveillance components, are directly involved in splicing [57,58,59,60,61,62,63]. Finally, splice-site mutations can cause Rrp6p-mediated nuclear retention of the unspliced RNAs and transcriptional down-regulation of the splicing-defective genes [43,64].

The exosome is a multi-subunit protein complex involved in RNA surveillance by degrading aberrantly processed RNAs and RNA processing intermediates [65]. Both nuclear and cytoplasmic exosomes have the same common core components, but are decorated with a variety of different peripheral proteins (such as Rrp6p, Dis3p, TRAMP and SKI complex) [66]. According to the current model, substrates of the nuclear exosome are recognized and subsequently recruited to the nuclear exosome by its cofactor, TRAMP complex [67,68,69]. The TRAMP complex is also a multi-protein complex comprising of the RNA helicase Mtr4p, a poly(A) polymerase (either Trf4p or Trf5p) and a zinc knuckle RNA binding protein (either Air1p or Air2p) [70]. The TRAMP complex cooperates with the nuclear exosome of eukaryotic cells and is involved in the 3' end processing of snoRNAs and ribosomal RNA. TRAMP complex is cotranscriptionally recruited to nascent RNA transcript [71], and physically interacts with spliced-out introns [72] and splicing factors [71,73], and thereby facilitates their degradation by the exosome. Deletion of TRAMP components leads to further accumulation of unspliced pre-mRNAs even in a yeast strain defective in nuclear exosome activity, suggesting a novel stimulatory role of TRAMP in splicing [71]. The cotranscriptional recruitment of TRAMP before or during splicing may function as a fail-safe mechanism to ensure the preparation for the subsequent targeting of spliced-out introns for rapid degradation by the nuclear exosome [71,73].

Consistent with the hypothesis above, recent study demonstrated that two shuttling SR proteins Gbp2p and Hrb1p are necessary for quality control of spliced mRNAs [74]. Gbp2p and Hrb1p stabilize the binding between TRAMP complex and spliceosome-bound transcripts [74]. Unspliced RNAs are retained in the nucleus and channeled to the TRAMP/exosome mediated degradation by Gbp2p and Hrb1p [74]. Taken together, Gbp2p and Hrb1p function as part of the fail-safe mechanism to ensure the cotranscriptional recruitment of TRAMP before or during splicing to prepare for the subsequent targeting of spliced-out introns to rapid degradation by the nuclear exosome. However, it remains unclear when the nuclear exosome and TRAMP are recruited and how they recognize unspliced pre-RNAs or spliced introns.

## 3. Spliceosome-Mediated Decay

**Figure 3:**
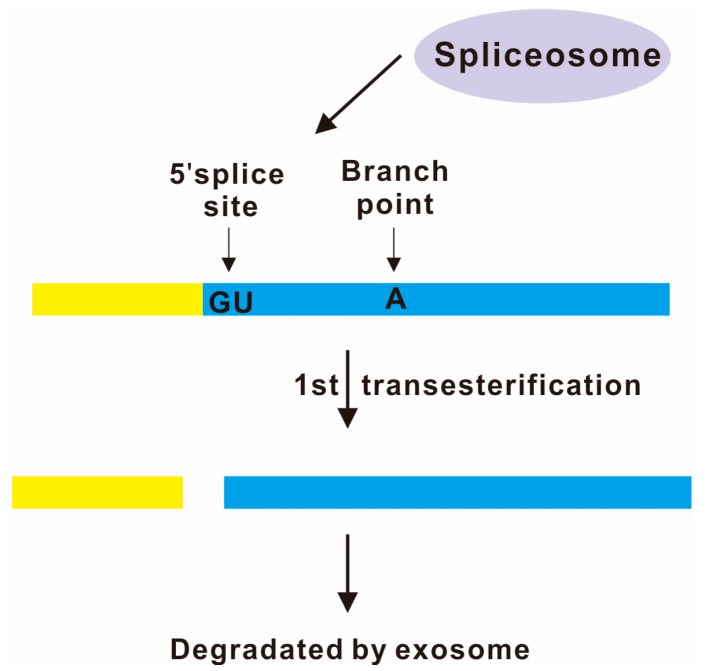
Many intronless mRNAs contain splice signals similar to 5' splice site and branch point. Spliceosome are recruited by the splice signals and catalyzes the first transesterification. Maybe due to lack of proper 3' splice site required for the canonical pre-mRNA splicing as shown in Figure 1, spliceosome only cleaves the intronless mRNA at the 5' splice site without proceeding to the second transesterification. The incompletely spliced products are degraded by the nuclear exosome. Ineffective transition from the first to the second step of splicing could also promote the pre-mRNA to nuclear degradation [75].

Spliceosome-mediated decay (SMD) was first proposed in 2013 when it was observed that the expression of ~1% of mRNAs without any intron were upregulated in the yeast cells defective with the splicing factor *PRP40* [75]. Spliceosome associates with those intronless mRNAs probably through the *cis*-elements similar to 5' splice site and branchpoint splice signals (Figure 3). The spliceosome endonucleolytically cleaves those intronless mRNA and the products are degraded by a nuclear RNA surveillance system [75]. The existence of SMD provided a plausible explanation for the coordinated regulation of expression levels of the homologous genes bromodomain factor *(BDF) 1* and *BDF2* in the yeast under different stress conditions [76]. Interestingly, the expression level of *BDF2* is also subjected to an additional layer of post-transcriptional control through RNase III-mediated decay (RMD) [77]. RNase III Rnt1p cleaves a stem-loop structure within the *BDF2* mRNA to down-regulate its expression [77]. The SMD and RMD pathways of the *BDF2* mRNA are differentially activated or repressed in specific environmental conditions [77]. The crosstalk between SMD and RMD pathways remain to be further explored.

## 4. Splicing and microRNA Processing

**Figure 4:**
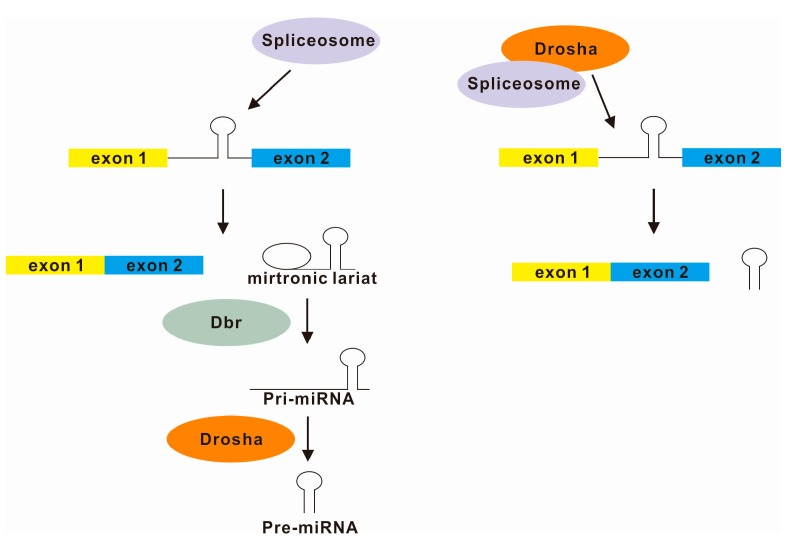
**Left panel**, according to the current model of mirtronic microRNAs biogenesis, spliced mirtronic lariat was first linearized by the debranching enzyme (Dbr) and then cleaved by Drosha; **Right panel**, recent studies suggested that splicing and microRNA processing are more closely associated than previously thought. Drosha is recruited to splice site with spliceosome as supraspliceosome [84,85]. Drosha may play a key role in the coordination of the regulation of mirtronic microRNAs biogenesis and splicing.

miRNAs are categorized as “intergenic” or “intronic” by their genomic locations. Large-scale bioinformatic analysis identified that many pre-microRNAs (miRNAs) are located in introns (named mirtrons) [78,79,80] or across exon-intron junctions [81]. As intronic miRNAs share common regulatory mechanisms with their host genes, the expression patterns of intronic miRNAs and their host genes are similar, while intergenic miRNAs are known to be transcribed as independent transcription units [82]. As shown in Figure 4, coupling between the splicing and microRNA processing machineries within a supraspliceosome context was proposed [83,84,85,86]. Supraspliceosome is a huge (21 MDa) nuclear ribonucleoprotein (RNP) complex in which numerous pre-mRNA processing steps take place [87]. Two key components of microRNA processing (the ribonuclease (RNase) III enzyme Drosha and the RNA binding protein DGCR8) and pre-miRNAs are co-sedimented with supraspliceosomes by glycerol gradient fractionation [85]. Other splicing factors such as serine/arginine-rich splicing factor 1 (SRSF1; Formerly SF2/ASF), heterogeneous nuclear ribonucleoprotein (hnRNP) A1 and K homology (KH) domain RNA binding protein (KSRP) have been proposed with moonlighting function in microRNA processing [88,89,90,91]. Processed pri-miRNAs are also found in supraspliceosomes [87]. Recent findings supported the model that the initiation of spliceosome assembly at the 5' splice site promotes microRNA processing by recruiting Drosha to intronic miRNAs [92]. Knockdown of U1 splicing factors globally reduces intronic miRNAs. It is consistent with the notion that the first step of the processing of mirtrons is splicing instead of microRNA processing and the debranched introns mimic the structural features of pre-miRNAs to enter the miRNA-processing pathway without Drosha-mediated cleavage [93]. Interestingly, Drosha may function as a splicing enhancer and promote exon inclusion [94]. Drosha binds to the exon and stimulates splicing in a cleavage-independent but structure-dependent manner [94]. To sum up, the expression of mirtrons is positively regulated by the splicing and microRNA processing.

Interestingly, some intronic miRNAs in humans can be transcribed independently of their host genes. The competition model between spliceosome and microRNA processing complex was proposed especially for miRNAs across exon-intron junctions [81,95]. It was suggested that nearby *cis*-elements and pre-miRNA secondary structure would interfere with splice site recognition [81,95]. In addition, inhibition of splicing by spliceostatin A upregulates the levels of the intronic miRNAs [85], whereas overexpression of Drosha increases the levels of the intronic and the exonic miRNAs [81]. These findings strongly supported that Drosha, instead of the miRNAs generated from canonical miRNA gene silencing pathway, directly represses the expression of genes by cleavage of the mRNAs [81].

## 5. Splicing and Circular RNAs

Circular RNAs are widely expressed noncoding RNAs and are generated cotranscriptionally by non-canonical mode of RNA splicing [32,83,96,97]. As mentioned above, during splicing, the spliceosome produces a free OH group at the 3' end of the intron. This free OH group attacks the phosphodiester bond between the downstream exon and intron. A debranching failure and “back-splicing” (a process in which downstream exons are spliced to upstream exons in reverse order [33,83,98,99,100]) produces a circular intronic long non-coding RNAs [101]. Recent deep sequencing studies have clearly revealed that thousands of circular RNAs generated from protein-coding genes in many organisms including human, and the number of circular RNAs per cell is far more than their linear protein-coding RNAs counterparts [83,102,103,104,105,106,107]. The accumulation of circular RNAs in cells may be attributed to the higher resistance of circular RNAs to endogenous exoribonucleases and hence their longer half-life [100,107,108].

Although circular RNAs are produced during splicing, the production of circular RNAs competes with canonical pre-mRNA splicing was also observed [96]. The production of these circular RNAs is mediated by intronic sequences [96,102,103,109]. A recent study demonstrated that the expression of a subset of circular RNA is regulated by the splicing factor muscleblind [96]. Therefore, circular RNAs may not only represent products of defective pre-mRNA splicing and nuclear RNA surveillance. They may actually be actively produced [34]. Interestingly, the production of circular RNAs seems to be responsible for a decline in the efficiency of canonical linear splicing. Circular RNAs accumulate in the nervous system and increase with age in *Drosophila* [110]. The mechanism and function of age-related modulation of circular RNA accumulation remain to be explored.

The function of most circular RNAs remains unclear, although their expression levels are closely related to diseases [105,111]. As circular RNAs are mainly found in the nucleus rather than the cytoplasm [103], and circular RNAs lack proper start and/or stop codons, it is unlikely that circular RNAs can code for proteins. However, a number of mechanisms of the regulatory potency of circular RNAs in gene expression are proposed. Certain circular RNAs function in regulating the expression of their host genes [103]. Circular RNAs accumulate at their sites of transcription, associate with elongation RNA polymerase II (RNAP II), and acts as a positive regulator of RNAP II transcription [103]. Some of these circular RNAs have been shown to act as molecular sponges by competing and/or sequestering miRNAs, and hence regulates miRNA level [112]. The potential function of circular RNAs in gene expression, their association with diseases in humans and their implications for therapeutic applications remains to be further explored [34,113].

## 6. Conclusions and Perspectives

In summary, the interactions between splicing and other RNA processing systems are more complicated and dynamic than we have ever thought. How does the exosome distinguish its targets splicing intermediates from the fully spliced RNAs? How is the expression of the selected splicing variants, intronic miRNAs and circular RNAs regulated through the coordination of the pre-RNA splicing and other RNA processing pathways? Those fundamental questions remain unaddressed. Through advances in technologies [114,115,116], development of new strategies [117,118,119,120,121,122,123], and establishment of databases for sharing information [124,125,126], hopefully those questions will be addressed in the near future.

## References

[1] Neverov A.D., Artamonova II, Nurtdinov R.N., Frishman D., Gelfand M.S., Mironov A.A. (2005). Alternative splicing and protein function. BMC Bioinform..

[2] Wu J.Y., Tang H., Havlioglu N. (2003). Alternative pre-mRNA splicing and regulation of programmed cell death. Regul. Altern. Splicing.

[3] Rino J., Carvalho T., Braga J., Desterro J.M., Luhrmann R., Carmo-Fonseca M. (2007). A stochastic view of spliceosome assembly and recycling in the nucleus. PLoS Comput. Biol..

[4] Montemayor E.J., Katolik A., Clark N.E., Taylor A.B., Schuermann J.P., Combs D.J., Johnsson R., Holloway S.P., Stevens S.W., Damha M.J. (2014). Structural basis of lariat RNA recognition by the intron debranching enzyme Dbr1. Nucleic Acids Res..

[5] Armakola M., Higgins M.J., Figley M.D., Barmada S.J., Scarborough E.A. (2012). Inhibition of RNA lariat debranching enzyme suppresses TDP-43 toxicity in ALS disease models. Nat. Genet..

[6] Hesselberth J.R. (2013). Lives that introns lead after splicing. Wiley Interdiscip. Rev. RNA.

[7] Baralle D., Baralle M. (2005). Splicing in action: Assessing disease causing sequence changes. J. Med. Genet..

[8] Chasin L.A. (2007). Searching for splicing motifs. Adv. Exp. Med. Biol..

[9] Fairbrother W.G., Yeh R.F., Sharp P.A., Burge C.B. (2002). Predictive identification of exonic splicing enhancers in human genes. Science.

[10] Krawczak M., Reiss J., Cooper D.N. (1992). The mutational spectrum of single base-pair substitutions in mRNA splice junctions of human genes: Causes and consequences. Hum. Genet..

[11] Cartegni L., Chew S.L., Krainer A.R. (2002). Listening to silence and understanding nonsense: Exonic mutations that affect splicing. Nat. Rev. Genet..

[12] Lim K.H., Ferraris L., Filloux M.E., Raphael B.J., Fairbrother W.G. (2011). Using positional distribution to identify splicing elements and predict pre-mRNA processing defects in human genes. Proc. Natl. Acad. Sci. USA.

[13] Wang J., Zhang J., Li K., Zhao W., Cui Q. (2012). SpliceDisease database: Linking RNA splicing and disease. Nucleic Acids Res..

[14] Kapustin Y., Chan E., Sarkar R., Wong F., Vorechovsky I., Winston R.M., Tatusova T., Dibb N.J. (2011). Cryptic splice sites and split genes. Nucleic Acids Res..

[15] Faustino N.A., Cooper T.A. (2003). Pre-mRNA splicing and human disease. Genes Dev..

[16] Hui J. (2009). Regulation of mammalian pre-mRNA splicing. Sci. China Ser. C Life Sci..

[17] Iborra S., Hirschfeld M., Jaeger M., Zur Hausen A., Braicu I., Sehouli J., Gitsch G., Stickeler E. (2014). Alterations in expression pattern of splicing factors in epithelial ovarian cancer and its clinical impact. Int. J. Gynecol. Cancer.

[18] Das S., Krainer A.R. (2014). Emerging functions of SRSF1, splicing factor and oncoprotein, in RNA metabolism and cancer. Mol. Cancer Res..

[19] Hickey C.J., Kim J.H., Ahn E.Y. (2014). New discoveries of old SON: A link between RNA splicing and cancer. J. Cell Biochem..

[20] Vaz-Drago R., Pinheiro M.T., Martins S., Enguita F., Carmo-Fonseca M., Custódio N. (2015). Transcription-coupled RNA surveillance in human genetic diseases caused by splice site mutations. Hum. Mol. Genet..

[21] Wood M.J., Gait M.J., Yin H. (2010). RNA-targeted splice-correction therapy for neuromuscular disease. Brain.

[22] Kole R., Leppert B.J. (2012). Targeting mRNA splicing as a potential treatment for duchenne muscular dystrophy. Discov. Med..

[23] Kole R., Krainer A.R., Altman S. (2012). RNA therapeutics: Beyond RNA interference and antisense oligonucleotides. Nat. Rev. Drug Discov..

[24] Ward A.J., Cooper T.A. (2010). The pathobiology of splicing. J. Pathol..

[25] Cooper T.A., Wan L., Dreyfuss G. (2009). RNA and disease. Cell.

[26] Wahl M.C., Will C.L., Luhrmann R. (2009). The spliceosome: Design principles of a dynamic RNP machine. Cell.

[27] Keren H., Lev-Maor G., Ast G. (2010). Alternative splicing and evolution: Diversification, exon definition and function. Nat. Rev. Genet..

[28] Black D.L. (2003). Mechanisms of alternative pre-messenger RNA splicing. Annu. Rev. Biochem..

[29] Stoilov P., Daoud R., Nayler O., Stamm S. (2004). Human tra2-β1 autoregulates its protein concentration by influencing alternative splicing of its pre-mRNA. Hum. Mol. Genet..

[30] Wang E.T., Sandberg R., Luo S., Khrebtukova I., Zhang L., Mayr C., Kingsmore S.F., Schroth G.P., Burge C.B. (2008). Alternative isoform regulation in human tissue transcriptomes. Nature.

[31] Johnson J.M., Castle J., Garrett-Engele P., Kan Z., Loerch P.M., Armour C.D., Santos R., Schadt E.E., Stoughton R., Shoemaker D.D. (2003). Genome-wide survey of human alternative pre-mRNA splicing with exon junction microarrays. Science.

[32] Salzman J., Gawad C., Wang P.L., Lacayo N., Brown P.O. (2012). Circular RNAs are the predominant transcript isoform from hundreds of human genes in diverse cell types. PLoS One.

[33] Yu C.Y., Liu H.J., Hung L.Y., Kuo H.C., Chuang T.J. (2014). Is an observed non-co-linear RNA product spliced in *trans*, in *cis* or just *in vitro*?. Nucleic Acids Res..

[34] Valdmanis P.N., Kay M.A. (2013). The expanding repertoire of circular RNAs. Mol. Ther..

[35] Zhang X.O., Wang H.B., Zhang Y., Lu X., Chen L.L., Yang L. (2014). Complementary sequence-mediated exon circularization. Cell.

[36] Vicens Q., Westhof E. (2014). Biogenesis of circular RNAs. Cell.

[37] Baejen C., Torkler P., Gressel S., Essig K., Soding J., Cramer P. (2014). Transcriptome maps of mRNP biogenesis factors define pre-mRNA recognition. Mol. Cell.

[38] Delan-Forino C., Tollervey D. (2014). Lighting up pre-mRNA recognition. Mol. Cell.

[39] Koodathingal P., Staley J.P. (2013). Splicing fidelity: DEAD/H-box ATPases as molecular clocks. RNA Biol.

[40] Yang F., Wang X.Y., Zhang ZM., Pu J., Fan Y.J., Zhou J., Query C.C., Xu Y.Z. (2013). Splicing proofreading at 5' splice sites by ATPase Prp28p. Nucleic Acids Res..

[41] Bonde M.M., Voegeli S., Baudrimont A., Seraphin B., Becskei A. (2014). Quantification of pre-mRNA escape rate and synergy in splicing. Nucleic Acids Res..

[42] Kawashima T., Pellegrini M., Chanfreau G.F. (2009). Nonsense-mediated mRNA decay mutes the splicing defects of spliceosome component mutations. RNA.

[43] Eberle A.B., Hessle V., Helbig R., Dantoft W., Gimber N., Visa N. (2010). Splice-site mutations cause Rrp6-mediated nuclear retention of the unspliced RNAs and transcriptional down-regulation of the splicing-defective genes. PLoS One.

[44] Niemela E.H., Oghabian A., Staals RH., Greco D., Pruijn G.J., Frilander M.J. (2014). Global analysis of the nuclear processing of transcripts with unspliced U12-type introns by the exosome. Nucleic Acids Res..

[45] Kawashima T., Douglass S., Gabunilas J., Pellegrini M., Chanfreau G.F. (2014). Widespread use of non-productive alternative splice sites in *Saccharomyces cerevisiae*. PLoS Genet..

[46] Chanfreau G.F. (2010). A dual role for RNA splicing signals. EMBO Rep.

[47] Davidson L., Kerr A., West S. (2012). Co-transcriptional degradation of aberrant pre-mRNA by Xrn2. EMBO J..

[48] Egecioglu D.E., Kawashima T.R., Chanfreau G.F. (2012). Quality control of *MATa1* splicing and exon skipping by nuclear RNA degradation. Nucleic Acids Res..

[49] Isken O., Maquat L.E. (2007). Quality control of eukaryotic mRNA: Safeguarding cells from abnormal mRNA function. Genes Dev..

[50] Chang Y.F., Imam J.S., Wilkinson M.F. (2007). The nonsense-mediated decay RNA surveillance pathway. Annu. Rev. Biochem..

[51] Shyu A.B., Wilkinson M.F., van Hoof A. (2008). Messenger RNA regulation: To translate or to degrade. EMBO J..

[52] Galy V., Gadal O., Fromont-Racine M., Romano A., Jacquier A., Nehrbass U. (2004). Nuclear retention of unspliced mRNAs in yeast is mediated by perinuclear Mlp1. Cell.

[53] Gencheva M., Lin T.Y., Wu X., Yang L., Richard C., Jones M., Lin S.B., Lin R.J. (2010). Nuclear retention of unspliced pre-mRNAs by mutant DHX16/hPRP2, a spliceosomal DEAH-box protein. J. Biol. Chem..

[54] Wery M., Ruidant S., Schillewaert S., Lepore N., Lafontaine D.L. (2009). The nuclear poly(A) polymerase and Exosome cofactor Trf5 is recruited cotranscriptionally to nucleolar surveillance. RNA.

[55] Vasiljeva L., Kim M., Terzi N., Soares L.M., Buratowski S. (2008). Transcription termination and RNA degradation contribute to silencing of RNA polymerase II transcription within heterochromatin. Mol. Cell.

[56] Schmid M., Jensen T.H. (2008). Quality control of mRNP in the nucleus. Chromosoma.

[57] Kress T.L., Krogan N.J., Guthrie C. (2008). A single SR-like protein, Npl3, promotes pre-mRNA splicing in budding yeast. Mol. Cell.

[58] Chen Y.C., Milliman E.J., Goulet I., Cote J., Jackson C.A., Vollbracht J.A., Yu M.C. (2010). Protein arginine methylation facilitates cotranscriptional recruitment of pre-mRNA splicing factors. Mol. Cell Biol..

[59] Lenasi T., Peterlin B.M., Barboric M. (2011). Cap-binding protein complex links pre-mRNA capping to transcription elongation and alternative splicing through positive transcription elongation factor b (P-TEFb). J. Biol. Chem..

[60] Garcia-Mayoral M.F., Hollingworth D., Masino L., Diaz-Moreno I., Kelly G., Gherzi R., Chou C.F., Chen C.Y., Ramos A. (2007). The structure of the *C*-terminal KH domains of KSRP reveals a noncanonical motif important for mRNA degradation. Structure.

[61] Golisz A., Sikorski P.J., Kruszka K., Kufel J. (2013). Arabidopsis thaliana LSM proteins function in mRNA splicing and degradation. Nucleic Acids Res..

[62] Wong C.M., Qiu H., Hu C., Dong J., Hinnebusch A.G. (2007). Yeast cap binding complex impedes recruitment of cleavage factor IA to weak termination sites. Mol. Cell Biol..

[63] Wong C.M., Tang H.M., Kong K.Y., Wong G.W., Qiu H., Jin D.Y., Hinnebusch A.G. (2010). Yeast arginine methyltransferase Hmt1p regulates transcription elongation and termination by methylating Npl3p. Nucleic Acids Res..

[64] Hessle V., von Euler A., Gonzalez de Valdivia E., Visa N. (2012). Rrp6 is recruited to transcribed genes and accompanies the spliced mRNA to the nuclear pore. RNA.

[65] Houseley J., Tollervey D. (2008). The nuclear RNA surveillance machinery: The link between ncRNAs and genome structure in budding yeast?. Biochim. Biophys. Acta.

[66] Synowsky S.A., van Wijk M., Raijmakers R., Heck A.J. (2009). Comparative multiplexed mass spectrometric analyses of endogenously expressed yeast nuclear and cytoplasmic exosomes. J. Mol. Biol..

[67] Stutz F., Izaurralde E. (2003). The interplay of nuclear mRNP assembly, mRNA surveillance and export. Trends Cell Biol..

[68] Vasudevan S., Peltz S.W. (2003). Nuclear mRNA surveillance. Curr. Opin. Cell Biol..

[69] Schmidt K., Butler J.S. (2013). Nuclear RNA surveillance: role of TRAMP in controlling exosome specificity. Wiley Interdiscip. Rev. RNA.

[70] LaCava J., Houseley J., Saveanu C., Petfalski E., Thompson E., Jacquier A., Tollervey D. (2005). RNA degradation by the exosome is promoted by a nuclear polyadenylation complex. Cell.

[71] Kong K.Y., Tang H.M., Pan K., Huang Z., Lee T.H., Hinnebusch A.G., Jin D.Y., Wong C.M. (2014). Cotranscriptional recruitment of yeast TRAMP complex to intronic sequences promotes optimal pre-mRNA splicing. Nucleic Acids Res..

[72] San Paolo S., Vanacova S., Schenk L., Scherrer T., Blank D., Keller W., Gerber A.P. (2009). Distinct roles of non-canonical poly(A) polymerases in RNA metabolism. PLoS Genet..

[73] Nag A., Steitz J.A. (2012). Tri-snRNP-associated proteins interact with subunits of the TRAMP and nuclear exosome complexes, linking RNA decay and pre-mRNA splicing. RNA Biol..

[74] Hackmann A., Wu H., Schneider U.M., Meyer K., Jung K., Krebber H. (2014). Quality control of spliced mRNAs requires the shuttling SR proteins Gbp2 and Hrb1. Nat. Commun..

[75] Volanakis A., Passoni M., Hector R.D., Shah S., Kilchert C., Granneman S., Vasiljeva L. (2013). Spliceosome-mediated decay (SMD) regulates expression of nonintronic genes in budding yeast. Genes Dev..

[76] Fu J., Hou J., Liu L., Chen L., Wang M., Shen Y., Zhang Z., Bao X. (2013). Interplay between *BDF1* and *BDF2* and their roles in regulating the yeast salt stress response. FEBS J..

[77] Alexandrov A., Colognori D., Shu M.D., Steitz J.A. (2012). Human spliceosomal protein CWC22 plays a role in coupling splicing to exon junction complex deposition and nonsense-mediated decay. Proc. Natl. Acad. Sci. USA.

[78] Kim Y.K., Kim V.N. (2007). Processing of intronic microRNAs. EMBO J..

[79] Rodriguez A., Griffiths-Jones S., Ashurst J.L., Bradley A. (2004). Identification of mammalian microRNA host genes and transcription units. Genome Res..

[80] Westholm J.O., Lai E.C. (2011). Mirtrons: MicroRNA biogenesis via splicing. Biochimie.

[81] Melamed Z., Levy A., Ashwal-Fluss R., Lev-Maor G., Mekahel K., Atias N., Gilad S., Sharan R., Levy C., Kadener S. (2013). Alternative splicing regulates biogenesis of miRNAs located across exon-intron junctions. Mol. Cell.

[82] Mattioli C., Pianigiani G., Pagani F. (2014). Cross talk between spliceosome and microprocessor defines the fate of pre-mRNA. Wiley Interdiscip. Rev. RNA.

[83] Kataoka N., Fujita M., Ohno M. (2009). Functional association of the Microprocessor complex with the spliceosome. Mol. Cell Biol..

[84] Shomron N., Levy C. (2009). MicroRNA-biogenesis and pre-mRNA splicing crosstalk. J. Biomed. Biotechnol..

[85] Agranat-Tamir L., Shomron N., Sperling J., Sperling R. (2014). Interplay between pre-mRNA splicing and microRNA biogenesis within the supraspliceosome. Nucleic Acids Res..

[86] Szweykowska-Kulinska Z., Jarmolowski A., Vazquez F. (2013). The crosstalk between plant microRNA biogenesis factors and the spliceosome. Plant Signal. Behav..

[87] Shefer K., Sperling J., Sperling R. (2014). The Supraspliceosome—A multi-task machine for regulated pre-mRNA processing in the cell nucleus. Comput. Struct. Biotechnol. J..

[88] Guil S., Caceres J.F. (2007). The multifunctional RNA-binding protein hnRNP A1 is required for processing of miR-18a. Nat. Struct. Mol. Biol..

[89] Wu H., Sun S., Tu K., Gao Y., Xie B., Krainer A.R., Zhu J. (2010). A splicing-independent function of SF2/ASF in microRNA processing. Mol. Cell.

[90] Michlewski G., Caceres J.F. (2010). Antagonistic role of hnRNP A1 and KSRP in the regulation of let-7a biogenesis. Nat. Struct. Mol. Biol..

[91] Trabucchi M., Briata P., Garcia-Mayoral M., Haase A.D., Filipowicz W., Ramos A., Gherzi R., Rosenfeld M.G. (2009). The RNA-binding protein KSRP promotes the biogenesis of a subset of microRNAs. Nature.

[92] Janas M.M., Khaled M., Schubert S., Bernstein J.G., Golan D., Veguilla R.A., Fisher D.E., Shomron N., Levy C., Novina C.D. (2011). Feed-forward microprocessing and splicing activities at a microRNA-containing intron. PLoS Genet..

[93] Ruby J.G., Jan C.H., Bartel D.P. (2007). Intronic microRNA precursors that bypass Drosha processing. Nature.

[94] Havens M.A., Reich A.A., Hastings M.L. (2014). Drosha promotes splicing of a pre-microRNA-like alternative exon. PLoS Genet..

[95] Mattioli C., Pianigiani G., Pagani F. (2013). A competitive regulatory mechanism discriminates between juxtaposed splice sites and pri-miRNA structures. Nucleic Acids Res..

[96] Ashwal-Fluss R., Meyer M., Pamudurti N.R., Ivanov A., Bartok O., Hanan M., Evantal N., Memczak S., Rajewsky N., Kadener S. (2014). circRNA biogenesis competes with pre-mRNA splicing. Mol. Cell.

[97] Memczak S., Jens M., Elefsinioti A., Torti F., Krueger J., Rybak A., Maier L., Mackowiak S.D., Gregersen L.H., Munschauer M. (2013). Circular RNAs are a large class of animal RNAs with regulatory potency. Nature.

[98] Wilusz J.E., Sharp P.A. (2013). Molecular biology. A circuitous route to noncoding RNA. Science.

[99] Wang Y., Wang Z. (2015). Efficient backsplicing produces translatable circular mRNAs. RNA.

[100] Lasda E., Parker R. (2014). Circular RNAs: Diversity of form and function. RNA.

[101] Yang L., Chen L.L. (2014). Competition of RNA splicing: Line in or circle up. Sci. China Life Sci..

[102] Liang D., Wilusz J.E. (2014). Short intronic repeat sequences facilitate circular RNA production. Genes Dev..

[103] Zhang Y., Zhang X.O., Chen T., Xiang J.F., Yin Q.F., Xing Y.H., Zhu S., Yang L., Chen L.L. (2013). Circular intronic long noncoding RNAs. Mol. Cell.

[104] Jeck W.R., Sharpless N.E. (2014). Detecting and characterizing circular RNAs. Nat. Biotechnol..

[105] Ghosal S., Das S., Sen R., Basak P., Chakrabarti J. (2013). Circ2Traits: A comprehensive database for circular RNA potentially associated with disease and traits. Front. Genet..

[106] Wang P.L., Bao Y., Yee M.C., Barrett S.P., Hogan G.J., Olsen M.N., Dinneny J.R., Brown P.O., Salzman J. (2014). Circular RNA is expressed across the eukaryotic tree of life. PLoS One.

[107] Starke S., Jost I., Rossbach O., Schneider T., Schreiner S., Hung L.H., Bindereif A. (2015). Exon circularization requires canonical splice signals. Cell Rep..

[108] Suzuki H., Tsukahara T. (2014). A view of pre-mRNA splicing from RNase R resistant RNAs. Int. J. Mol. Sci..

[109] Jeck W.R., Sorrentino J.A., Wang K., Slevin M.K., Burd C.E., Liu J., Marzluff W.F., Sharpless N.E. (2013). Circular RNAs are abundant, conserved, and associated with ALU repeats. RNA.

[110] Westholm J.O., Miura P., Olson S., Shenker S., Joseph B., Sanfilippo P., Celniker S.E., Graveley B.R., Lai E.C. (2014). Genome-wide analysis of Drosophila circular RNAs reveals their structural and sequence properties and age-dependent neural accumulation. Cell Rep..

[111] Lukiw W.J. (2013). Circular RNA (circRNA) in Alzheimer’s disease (AD). Front. Genet..

[112] Hansen T.B., Jensen T.I., Clausen B.H., Bramsen J.B., Finsen B., Damgaard C.K., Kjems J. (2013). Natural RNA circles function as efficient microRNA sponges. Nature.

[113] Bak R.O., Hollensen A.K., Mikkelsen J.G. (2013). Managing microRNAs with vector-encoded decoy-type inhibitors. Mol. Ther..

[114] Ozsolak F., Milos P.M. (2011). RNA sequencing: Advances, challenges and opportunities. Nat. Rev. Genet..

[115] Sorenson M.R., Stevens S.W. (2014). Rapid identification of mRNA processing defects with a novel single-cell yeast reporter. RNA.

[116] Marinov G.K., Williams B.A., McCue K., Schroth G.P., Gertz J., Myers R.M., Wold B.J. (2014). From single-cell tocell-pool transcriptomes: Stochasticity in gene expression and RNA splicing. Genome Res..

[117] Schamberger A., Orban T.I. (2014). Experimental validation of predicted mammalian microRNAs of mirtron origin. Methods Mol. Biol..

[118] Wang Z., Rolish M.E., Yeo G., Tung V., Mawson M., Burge C.B. (2004). Systematic identification and analysis of exonic splicing silencers. Cell.

[119] Barash Y., Garcia J.V. (2014). Predicting alternative splicing. Methods Mol. Biol..

[120] Yadav A.R., Mace C.R., Miller B.L. (2014). Examining the interactions of the splicing factor MBNL1 with target RNA sequences via a label-free, multiplex method. Anal. Chem..

[121] Hsu J.B., Huang K.Y., Weng T.Y., Huang C.H., Lee T.Y. (2014). Incorporating significant amino acid pairs and protein domains to predict RNA splicing-related proteins with functional roles. J. Comput. Aided Mol. Des..

[122] Thompson B.A., Martins A., Spurdle A.B. (2015). A review of mismatch repair gene transcripts: Issues for interpretation of mRNA splicing assays. Clin. Genet..

[123] Hoffmann S., Otto C., Doose G., Tanzer A., Langenberger D., Christ S., Kunz M., Holdt L.M., Teupser D., Hackermüller J. (2014). A multi-split mapping algorithm for circular RNA, splicing, trans-splicing and fusion detection. Genome Biol.

[124] Sinha R., Lenser T., Jahn N., Gausmann U., Friedel S., Szafranski K., Huse K., Rosenstiel P., Hampe J., Schuster S. (2010). TassDB2—A comprehensive database of subtle alternative splicing events. BMC Bioinform..

[125] Zhang Y., Chen K., Sloan S.A., Bennett M.L., Scholze A.R., O’Keeffe S., Phatnani H.P., Guarnieri P., Caneda C., Ruderisch N. (2014). An RNA-sequencing transcriptome and splicing database of glia, neurons, and vascular cells of the cerebral cortex. J. Neurosci..

[126] Hatje K., Kollmar M. (2014). Kassiopeia: A database and web application for the analysis of mutually exclusive exomes of eukaryotes. BMC Genomics.

